# Dental Caries in Adult Patients with Rheumatoid Arthritis—A Systematic Review

**DOI:** 10.3390/jcm12124128

**Published:** 2023-06-19

**Authors:** Deborah Kreher, Bero Luke Vincent Ernst, Dirk Ziebolz, Rainer Haak, Thomas Ebert, Gerhard Schmalz

**Affiliations:** 1 Department of Cariology, Endodontology and Periodontology, University of Leipzig, 04109 Leipzig, Germany; deborah.kreher@medizin.uni-leipzig.de (D.K.); bero.ernst@medizin.uni-leipzig.de (B.L.V.E.); dirk.ziebolz@medizin.uni-leipzig.de (D.Z.); rainer.haak@medizin.uni-leipzig.de (R.H.); 2 Medical Department III—Endocrinology, Nephrology, Rheumatology, University of Leipzig, 04109 Leipzig, Germany; thomas.ebert@medizin.uni-leipzig.de

**Keywords:** oral health, dental caries, rheumatoid arthritis

## Abstract

Patients suffering from rheumatoid arthritis (RA) are repeatedly affected by oral diseases or complaints, including xerostomia, periodontitis and dental caries. The aim of this systematic review was the evaluation of caries prevalence and/or incidence in patients with RA. Within this review, there is a systematic search of the literature based on PubMed, Web of Science and Scopus. Two independent researchers performed the search in February 2023. The search terms were “dental caries” AND “rheumatoid arthritis”. Additionally, a manual search completed the review process. Studies on adult patients (age ≥ 18 years) only suffering from RA were included. Studies had to explicitly report on the prevalence or incidence of dental caries. The respective studies were checked regarding suitability and, if they were eligible, analyzed qualitatively. A quality appraisal was performed for all of the analyzed studies. A total of 336 studies were detected, of which 16 studies met the in- and exclusion criteria. The sample sizes of the clinical investigations ranged between 13 and 1337 participants. Twelve studies evaluated a healthy control group. In 8/12 studies, a significant difference in the prevalence/incidence of caries was found between RA patients and controls. The majority of the studies applied the decayed (DT), missing and filled teeth index (DMFT) for the diagnosis of caries. On average (mean value), 0.8 to 5.79 carious teeth per patient were reported across the studies. There was no information on the stadium, activity or location of caries (e.g., root caries) in any study. Quality appraisal revealed a moderate quality for most studies. In conclusion, caries prevalence was heterogeneous across studies, while a higher caries prevalence was repeatedly reported in RA patients against controls. Further research regarding dental caries in RA appears recommendable; multidisciplinary, patient-centered dental care for patients with RA should be fostered to improve patients’ dental health status.

## 1. Introduction

Globally, dental caries is highly prevalent and, therefore, one of the most frequently occurring chronic diseases [1,2], potentially affecting people in any age group. From childhood to retirement age, caries develops in different areas of the teeth. Children and (young) adults often develop caries at the crowns of teeth, while elderly individuals are concerned by root caries lesions [3]. Caries can present itself as anything from a small white spot to a deep cavity in the tooth surface, potentially leading to tooth decay, pain, acute exacerbation and tooth loss [3]. Its progression lasts several years and needs the accumulation of biofilm on teeth. Furthermore, there is a complex interaction between potentially cariogenic bacteria and fermentable carbohydrates, as well as various host factors including the quantity and quality of saliva [4]. A wide range of risk factors is known for the development of caries lesions. These include oral hygiene and carbohydrate intake, but several systemic diseases and associated symptoms can also be related to caries development and progression [5].

In this context, dry mouth/xerostomia, in particular, is an important factor influencing the occurrence of dental caries [6]. For example, it is generally known that especially patients with rheumatoid arthritis suffer from xerostomia [7]. Rheumatoid arthritis (RA) is comparably frequent among rheumatic diseases and clinically relevant with a global prevalence of 0.5–1% [8,9,10]. It is already known that patients with RA have a higher risk for developing infections [11]. Therefore, untreated dental diseases can be risk factors for systemic infections, where RA patients are particularly at risk due to their immunosuppressive medication [12]. Some studies in the existing literature show an association between dental caries and RA [13,14]. Moreover, patients with RA often suffer from more severe periodontitis [15]. The exposition of root surfaces caused by periodontal recession might, therefore, be an additional risk factor for the manifestation of caries in those individuals.

The evidence accumulated in this area of research in recent years shows some heterogeneity. Similarly, it remains unclear whether patients with RA suffer from root caries or specific lesions. Additionally, the assessment of caries or its diagnostic in the different studies appears different. Hence, the current systematic review aimed to evaluate the prevalence and/or incidence of dental caries in adults with RA. In the course of this, characteristics and results of the available studies were evaluated. Furthermore, a comparison of the caries prevalence between patients with RA and healthy control individuals was considered, if available. Moreover, an appraisal of the quality of the included studies was applied. It was hypothesized that patients with rheumatoid arthritis show a higher caries prevalence and/or incidence than healthy (non-RA) individuals.

## 2. Methods

This systematic review followed the Preferred Reporting Items for Systematic Reviews and Meta-Analyses (PRISMA) guidelines [16].

### 2.1. Central Issue of the Systematic Review

The PICO question of this review (patients, intervention, comparison, and outcome) was if adults with RA have a higher prevalence and/or incidence of caries than healthy individuals. For this, patients were only adults with diagnosed RA. There was no assessment of an intervention. If applicable, a healthy (non-RA) control group was used for comparison. In the end, the respective caries assessments were recorded as outcomes. It was hypothesized that adults with RA would present a higher caries prevalence and/or incidence than healthy individuals.

### 2.2. Eligibility Criteria

As inclusion criteria for the current systematic review, the exclusive examination of adult patients (age >18 years) suffering from RA was formulated. Therefore, the RA diagnosis was required to have been confirmed by a specialized physician, considering the respective diagnosis criteria. If data of population-based cohort studies or registers were reported, it was assumed that a qualified specialist had checked and approved the RA diagnoses in those patients. Another mandatory criterion was the clinical assessment of dental caries (prevalence or incidence), e.g., decayed teeth (DT) or decayed-missing-filled-teeth index (DMFT). An experienced examiner was required to have made the caries diagnosis. Studies without an English full text were excluded from the analysis. Further in- and exclusion criteria did not exist.

### 2.3. Search Strategy

Two independent individuals performed the literature search based on the databases of PubMed, Web of Science and Scopus in February 2023. The search terms used were “dental caries” AND “rheumatoid arthritis”. A manual search completed the process, whereby citations of the included articles were checked based on their respective reference lists. Grey literature was not explicitly searched within the systematic review process. After the systematic search, the studies were verified for their eligibility.

### 2.4. Data Extraction

From the included studies, several pieces of information were extracted during the review process:

- Publication year, study type, country;
- Sample size, sex, age, disease duration;
- Caries (e.g., decayed teeth (DT) or decayed-missing-filled-teeth index (DMFT), tooth loss/remaining teeth, oral hygiene parameters);
- Laboratory parameters (if applicable): c-reactive protein (CRP), erythrocyte sedimentation rate (ESR), disease activity score (DAS-28), rheumatoid factor (RF);
- Salivary parameters: salivary flow and pH-value;
- Bacterial metabolism (if applicable);
- Presence of a control group, sex, age.

Two independent reviewers conducted the systematic search, study selection and qualitative analysis. If any disputes occurred during the process, they were discussed with the senior author first and afterwards, if necessary, discussed and resolved among all the authors.

### 2.5. Quality Appraisal

A checklist, containing 11 items to evaluate the quality of included studies, i.e., the list for cross-sectional investigations by the Agency for Healthcare Research and Quality (AHRQ) [17], was used in this systematic review. “No” and “unclear” answers were scored 0 points and “yes” answers were scored 1 point for each question to determine a total score for the assessment. Sum scores of 0–3 indicated low quality, and 4–7 points implied moderate quality, while 8–11 points indicated high quality of the respective study. The appraisal was conducted independently by the first authors (D.K. and B.E.) and the senior author (G.S.). Disagreements were discussed and resolved among the authors.

## 3. Results

### 3.1. Systematic Search Results

**Figure 1:**
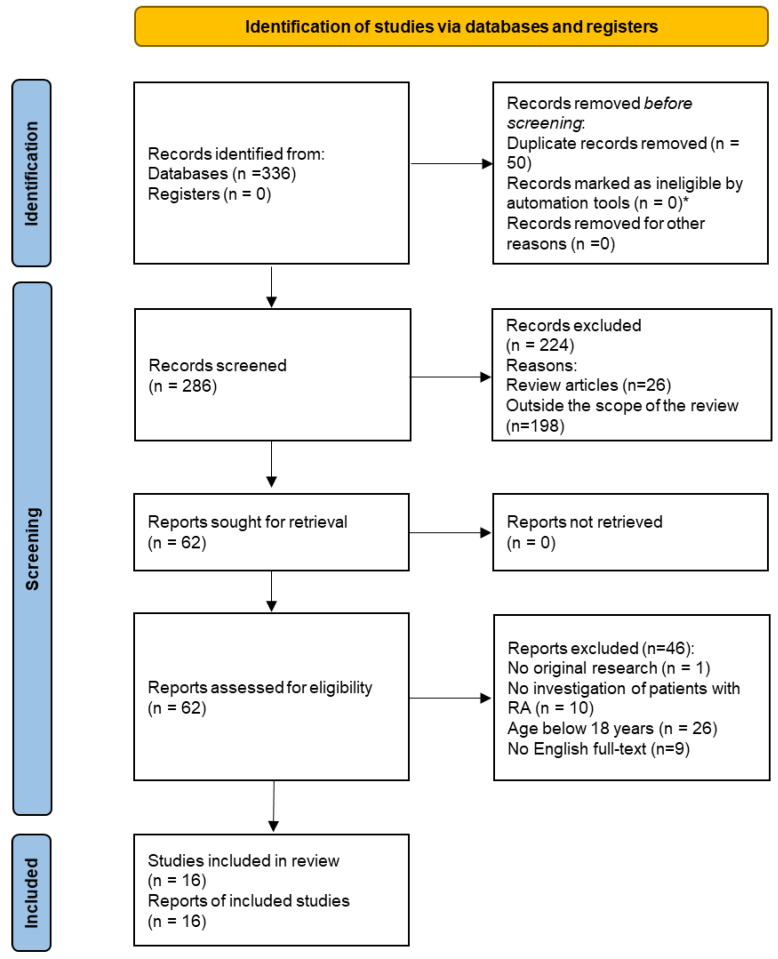
PRISMA flow diagram. * No automation tools were used.

According to the PRISMA statement [16], Figure 1 illustrates the search findings. The systematic search, supplemented by the manual search, identified 336 studies. Due to duplication, 50 studies were excluded, and another 224 studies were excluded during the subsequent screening. A total of 198 studies were off-topic, and in 26 cases, the studies were systematic reviews. After screening the records, 62 full texts were assessed for eligibility. A total of 46 of these studies were excluded, whereby 10 studies were also found to be off-topic, and in 26 studies, the participants were under 18 years of age. Moreover, in nine cases there was no English full text available. In total, 16 studies were included in the qualitative analysis (Figure 1).

### 3.2. Study Characteristics

The included studies were performed in 11 different countries. The sample sizes of the clinical investigations ranged between 13 and 1337 RA participants. Twelve studies examined a healthy control group for comparison. The study type, as well as the mean age, sex and disease duration of the participants, is shown in Table 1.

### 3.3. Oral Health Record and Findings

The studies which were included in the current systematic review showed heterogeneous oral examinations. Most studies assessed the caries burden using the index of decayed, missing and filled teeth (DMFT). All but five studies examined DMFT or decayed teeth (DT). The mean value of decayed teeth ranged between 0.8 and 5.79 carious teeth (DT) across the studies (Table 2). Six of the included studies also examined oral hygiene parameters, including the plaque index (PI), community periodontal index (CPI) and simplified oral hygiene index (SOHI). As shown in Table 2, there is a detailed overview of the examined oral health parameters and results. No study examined caries activity or the location of caries (e.g., the crown of the tooth or root surface).

Table 3 shows the prevalence of caries in RA patients compared with their respective control groups. In eight of twelve studies, a significant difference in caries prevalence/incidence was confirmed between RA patients and controls. Therefore, six of these studies detected worse caries burdens in individuals with RA (Table 3). The results are partially significant but heterogeneous between the different studies.

### 3.4. Quality Assessment

Quality appraisal according to AHRQ criteria showed that the majority of the included studies were evaluated as being of moderate quality (Table 4).

## 4. Discussion

A total of 16 studies from 12 different countries were qualitatively analyzed in the current review. These studies had a range in sample size between 13 and 1337 RA participants, indicating a heterogeneity across the investigations. A total of 12 of those 16 studies had a control group, and 8/12 showed a significant difference in caries prevalence between the RA patients and the control group. The majority of the studies investigated the presence of caries with DMFT or DT. In several studies, the caries prevalence was indicated by the investigators, and in one study, caries was detected only with a panoramic dental x-ray.

The hypothesis was formed that patients with RA would present a higher caries prevalence and/or incidence compared to healthy controls. After evaluation of the 16 studies, it was shown that the hypothesis can be confirmed because of the seven studies which demonstrated a significantly increased caries prevalence in RA patients. Only one study showed the opposite. Actually, a higher caries prevalence was displayed in the healthy control group [30]. However, it must be highlighted that there were several studies comparing RA and control patients which had a low sample size, and, therefore, their statistical power remains questionable (see Table 3). Mostly, the included studies appropriated the DMFT-index for clinical examinations. This index is regularly and repeatedly used in clinical studies for swift dental check-ups. Therefore, DT is applied to document carious lesions with cavitation of the tooth surfaces [31]. The disadvantage of this index is that early lesions (white spots or brown spots) are not recorded. Furthermore, the location and extent, or even the activity, of carious lesions are not taken into account. Similarly, as for RA patients, the literature shows that individuals with different rheumatic diseases suffer from a high oral disease burden, leading to an increased need of attention in dental practice and preventive measures [32]. Children and adolescents with juvenile idiopathic arthritis were found to suffer from different oral complaints, although an increased caries prevalence was not confirmed within a multilevel analysis [33]. Rheumatically diseased patients with oral symptoms, especially reduced salivary flow such as in Sjogren syndrome, have a high caries burden [34]. Additionally, patients with diffuse cutaneous systemic sclerosis show an increased caries prevalence, which is correlated with disease characteristics [35]. Overall, patients with rheumatic disease appear to show a high caries burden, although this differs between the various entities. In this context, a high caries prevalence in individuals with RA seems plausible.

One explanation for the increased incidence and prevalence of caries in patients with RA appears to be physical dysfunction and associated problems with oral hygiene. A study from 1992 already showed that patients with RA suffered from reduced physical function in their arms and hands. Participants of this study reported that hand problems would affect oral hygiene [19]. However, no correlation was found between physical dysfunctions and tooth loss by the investigators [19]. Another study from the working group of Mehdipour confirmed those findings and, additionally, mentioned that reasons for higher DMFT could be decreased salivary flow, increased salivary acidity, emergence of microorganisms and generalized osteoarthritis, specifically in the fingers and arms [14]. The study by Äyräväinen et al. in 2018 compared patients with early untreated RA (EURA) and patients with chronic RA (CRA), as well as a healthy control group [13]. The investigators showed that the oral health of RA patients was worse compared with the control group, and caries indices were associated with the activity of RA. Patients with a higher activity of RA showed increasing caries scores (DMFT, DT) [13]. This was the only study which carried out an intervention and a follow-up. The group of Äyräväinen also showed that EURA patients brushed their teeth less often than chronic RA patients did at the baseline, while after 16 months a significant improvement in the oral hygiene of the EURA patients was determined [13]. Furthermore, the activity of DAS-28 increased with higher caries indices, which might indicate an association between disease activity and caries [13]. Accordingly, the relationship between dental caries and RA-related parameters could be of certain interest. An Iranian study which was included in the current systematic review showed that caries was in an inverse relationship with the rheumatoid factor (RF) [23]. Taken together, disease severity, activity and disease-related disability might be one plausible explanation for the increased caries burden on individuals with RA.

Another potential issue is the saliva of patients with RA. The study of Risheim et al. investigated salivary flow and oral sugar clearance in patients suffering from RA and dry mouth. The study group demonstrated that rheumatic patients presented a decreased salivary flow (median unstimulated: 0.09 mL/min and stimulated: 0.9 mL/min). On the other hand, those patients with a long oral sugar clearance revealed more root caries lesions, lower resting salivary flow and higher salivary counts of mutans streptococci [36]. Furthermore, patients with RA have repeatedly shown xerostomia [7], which is an important risk factor for caries, as it reduces the potential of remineralization [6]. Additionally, a case–control study investigated salivary immunoglobulin A in RA patients and controls without finding a statistical significance between those two groups [37]. Altogether, the quantity and quality of saliva appears to affect caries in individuals with RA. However, only a minority of the studies which were included in this systematic review performed salivary analyses [13,18,26,29].

Another issue of potential relevance is the patient’s perspective; therefore, the oral health-related quality of life (OHRQoL) could play a potential role. A recent systematic review found a reduced OHRQoL in RA patients; however, this was often caused by disease-related and psychosocial factors [38]. In this context, it has been explained that chronically diseased patients can undergo a kind of response shift, while the perception of the burden of their general disease is so high that other issues, such as oral hygiene and oral health, might be upstaged [39]. Accordingly, a neglect of oral health behavior by individuals with RA could be an additional explanation for their increased caries prevalence.

Generally, it must be stated that several issues require consideration with regard to the included studies. The vast majority used DMFT, which is well-established for clinical studies [31]. However, this index does not include any information on the caries localization (crown or root) or the caries stadium (initial lesion, large lesion affecting the pulp chamber, etc.). As described previously in a systematic review on dental caries in patients with renal replacement therapy, improved caries diagnostics should be applied in such cohorts of at-risk patients, for example, by using extended clinical or adjunctive diagnostics [40]. Additionally, the heterogeneity and quality of the studies, which was moderate in most cases, underline the need for improvements in the research on dental caries in RA patients.

As a further issue, it is well-documented that RA is in a bidirectional relationship with periodontitis [15,41,42]. Therefore, host factors (the immune system) and bacteria were documented to link both diseases; the potential periodontal pathogen *Porphyromonas gingivalis* was especially found to increase RA-related inflammation, especially via citrullination of proteins [43]. Similarly, *Aggregatibacter actinomycetemcomitans*, another potential periodontal pathogenic bacterium, can be involved in the interplay between periodontitis and RA [44]. Additionally, RA can lead to changes in the jawbone and temporomandibular joint [45]. All of these issues could also affect caries development and progression. In this context, common acquired risk factors exist between caries and periodontitis, which might support the increased caries risk in patients with RA [46].

The strengths and limitations of this study are as follows. The current systematic review followed the PRISMA guidelines, while a clear PICO question was also formulated. Two independent reviewers performed the systematic search and the quality appraisal. Nevertheless, there are certain limitations, which are discussed in the following sentences. Studies from a variety of countries with various patient populations were reviewed. Therefore, dental examinations were performed under different conditions. No separate new examination took place when the results from national health studies were used. Hence, this limits the generalizability of the data. Besides this limitation, studies with and without a healthy control group were included. While for a definitive confirmation of the hypothesis of this review, a control group would be needed, it was decided to include studies without a control group to increase the number of included studies and, thus, the ability to draw conclusions. This was performed similarly to previous studies [40,47]. Most studies showed a moderate quality (Table 4). It should be mentioned that none of the studies except one conducted an intervention or follow-up. Only half of the studies included laboratory values or saliva examinations in their investigations. Considering the lack of differentiated caries diagnostics or any RA-related parameters, the available evidence remains limited. Therefore, in summary, there is a lack of homogeneity regarding the study designs, methods and populations. Additionally, a couple of studies examined a small sample. Furthermore, there is a lack of explanation for bias across the studies. Therefore, further well-performed clinical studies appear to be needed in the field to strengthen the body of evidence. The missing registration of this systematic review must be seen as another limitation.

## 5. Conclusions

In patients with RA, caries prevalence was heterogeneous across the studies, while an increased caries prevalence in RA patients against controls was repeatedly reported, indicating a higher prevalence of dental caries in individuals with RA. Different reasons appear probable, including salivary flow and composition, disease activity and physical disability. There still appears to be a lack of evidence and heterogeneity of the literature. Nevertheless, multi-disciplinary dental care of RA patients needs to be fostered to improve their oral situation.

## Figures and Tables

**Table 1:** Included examinations and their study characteristics. Results of age and disease duration are given as mean value ± standard deviation or mean value (range).

Author, Year	Country	No. of Patients	Study Type	Subjects’ Mean Age in Years	Treatment Time	Male (%)	Control Group (n, Age, Sex (%))
Helenius et al., 2005 [18]	Finland	24	monocentric	49.4 (11.5)	10.5 (2.6) years	8.3% men	24, 44.6 (12.7) years, 8.3% men
Arneberg et al., 1992 [19]	Southeastern Norway	125	monocentric	44–56 years (city of Oslo: 52 ± 3; Oslo suburbs: 51 ± 4; rest of southeastern Norway: 51 ± 3)	4–9 years: 26.4%, 10–19 years: 40.8%, 20–42 years: 32.8%	16.8% men	n/a
Descamps et al., 2020 [20]	France	223	monocentric retrospective	54.4 ± 10.9	8.9 ± 8.6 years	20.6% men	n/a
Gonzales-Chavez et al., 2019 a [21]	Mexico	30	monocentric	44.30 ± 13.50	n/a	7% men	30, 41.7 ± 12.25 years, 20% men
Mehdipour et al., 2022 [14]	Iran	45	cross-sectional descriptive analytical	53.5 ± 7.99	mean duration 5.82 ± 6.1 years (minimum 6 months, maximum 25 years)	16% men	45, 53.9 ± 7.55 years, % of men not clear
Gonzales-Chavez et al., 2019 b [22]	Mexico	62	monocentric descriptive observational	51 (18–72)	n/a	13%men	n/a
Äyräväinen et al., 2018 [13]	Finland	81 (EURA: n = 53, CRA: n = 28)	monocentric	EURA: 52 (22–78), CRA: 54 (20–64)	n/a	EURA: 15.1% men, CRA: 17.9% men	43, 56 (30–82) years, 11.6% men
Almasi et al., 2021 * [23]	Iran	118	monocentric case-control study	51.49 ± 10.91	9.15 ± 7.88	17.8% men	118 *
Kim et al., 2019 [24]	South Korea	157	data from fifth and sixth Korea National Health and Nutrition Examination Surveys	56.3 ± 1.4 years	n/a	22.3% men	20, 140, 43.9 ± 0.2 years, 42.5% men
Kroese et al., 2022 [25]	The Netherlands	150 (early rheumatoid arthritis n = 50, risk of RA n = 50)	cross-sectional (part of larger longitudinal cohort study)	RA: 52.1 (13.2) years, risk of RA: 51.4 (10.3) years	<1 year	RA: 22% men, risk of RA: 24% men	50, 51.2 (11.0) years, 24% men
Martinez-Martinez et al., 2019 [10]	Mexico	80	cross-sectional prospective study	46 ± 8 (rage: 32–60) years	≥5 years but <15 years	8% men	80, 46 ± 8 (rage: 32–60) years, 8% men
Silvestre-Rangil et al., 2016 [26]	Spain	73	prospective cross-sectional case-controlled study	53.3 ± 12.1 years	n/a	28.7% men	73, 52.6 ± 11.2 years, 32.8% men
Juan et al., 2022 [27]	Taiwan	1337	secondary cohort analysis with data from Taiwan’s National Health Insurance Research Database	53.2 (SD 13.4) years	newly diagnosed	22.6% men	13,370, 53.2 (SD 13.4) years, 22.6% men
Mok et al., 2022 [28]	Hong Kong	238	monocentric cross-sectional	58.8 ± 10.8 years	15.1 ± 11.0 years	6.3% men	n/a
Sánchez-Medrano et al., 2021 [29]	Mexico	13	descriptive pilot study	45 (SD 8.0) years	newly diagnosed	7% men	16, 49 (SD 10) years, 50% men
de Pablo et al., 2007 [30]	USA	103	cross-sectional survey: data from the third National Health Nutrition Examination Survey (USA)	73 ± 8.3 years	n/a	43% men	4358, 72 ± 8.1 years, 49% men

* No result tables with numerical values available. n/a: not applicable, RA: rheumatoid arthritis, EURA: early untreated rheumatoid arthritis, CRA: chronic rheumatoid arthritis.

**Table 2:** Oral conditions, dental caries and selected further parameters in the included examinations.

Author, Year	Tooth Loss, Remaining Teeth, Dentures	Caries	Oral Hygiene Parameters	Laboratory Parameters				Saliva Parameters		Bacterial-/Metabolism
				CRP (mg/L)	DAS-28	ESR (mm/h)	RF	Saliva Flow Rate	pH	
Helenius et al., 2005 [18]	n/a	Prevalence: RA: 75% in patients with missing teeth, 17% in patients with caries at clinical examination; C: 53% in patients with missing teeth, 22% in patients with caries at clinical examination	CPI (RA: 2 = 25%, 3 = 38%, 4 = 33%; C: 2 = 74%, 3 = 25%, 4 = 1%)	n/a	n/a	n/a	n/a	Resting flow: decreased flow RA: 8%, C: 0%; stimulated flow: decreased flow RA: 8%, C: 0%	low pH: RA: 17%; C: 4%	Mutans streptococci (>10^5^ CFU/mL): RA: 8%, C: 12%; Lactobacilli (>10^6^ CFU/mL): RA: 4%, C: 6%; positive yeast count: RA: 50%, C: 22%; A.a. pos: RA: 8%, C: 6%; P.g. pos: RA: 13%, C: 16%; P.i. pos: RA: 63%, C: 75%; P.n. pos: RA: 63%, C: 53%; B.f. pos: RA: 0%, C: 3%
Arneberg et al., 1992 [19]	mean number: 25	Caries problems: 0–2 cavities/yr: 78.4%, 3–5 cavities/yr: 10.4%, >5 cavities/yr: 5.6%	n/a	n/a	n/a	n/a	n/a	n/a	n/a	n/a
Descamps et al., 2020 [20]	n/a	Panoramic dental X-ray: 11.8% had dental caries	n/a	high CRP level (<5 mg/L): 63.5%	5.5 ± 2.6	n/a	77.4%	n/a	n/a	n/a
Gonzales-Chavez et al., 2019 a [21]	n/a	Missing: RA: 6.90 ± 5.77; C: 3.03 ± 2, cavities: RA: 13.46 ± 5.48, C: 4.90 ± 6.55; slight cavities: RA: 4.30 ± 4.39, C: 2.33 ± 3.91; moderate cavities: RA: 6.83 ± 5.25, C: 1.96 ± 3.48; advanced: RA: 2.33 ± 3.45, C: 0.6 ± 1.88	SOHI (RA: 3.29 ± 1.73; C: 1.51 ± 1.52)	n/a	n/a	n/a	n/a	n/a	n/a	n/a
Mehdipour et al., 2022 [14]	n/a	DMFT RA: 18.87 ± 6.76, C: 11.11 ± 5.90; DT: RA: 3.84 ± 3.90, C: 1.93 ± 1.86	n/a	yes *	yes *	yes *	n/a	n/a	n/a	n/a
Gonzales-Chavez et al., 2019 b [22]	n/a	missing: 73.8%, caries: 98.3% (initial: 70%, moderate: 91.7%, advanced: 73.8%), restored: 59%	OHI-S (3.2 (0.2–6))	10.9 (0.2–86.6)	4.1 (1–6.2)	33 (10–78)	82% positive	n/a	n/a	n/a
Äyräväinen et al., 2018 [13]	EURA: baseline: 27 (23–28), follow up: 27 (22–28); CRA: baseline: 27 (22–28), follow up: 27 (22–28), control: 27 (25–28)	DMFT: EURA: baseline: 19 (12–23), follow up: 20 (13–24); CRA: baseline: 19 (12–23), follow up: 19 (12–23), control: 17 (10–21)	n/a	EURA: baseline: 6 (3–14), follow up: 3 (2–6); CRA: baseline: 18 (5–30), follow up: 7 (2–19)	EURA: baseline: 4.0 (3.2–4.8), follow up: 2.4 (1.7–2.9); CRA: baseline: 4.1 (3.0–4.9), follow up: 3.1 (2.0–3.9)	EURA: baseline: 20 (11–34), follow up: 9 (5–16); CRA: baseline: 20 (9–46), follow up: 15 (5–31)	EURA: 79.2%; CRA: 69.2%; control: 8.1%	ml/ 5 min: unstimulated: EURA baseline: 1.0 (0.5–1.5), follow up: 1.4 (0.8–2.2); CRA baseline: 1.2 (0.7–2.0), follow up: 1.7 (0.8–2.3); stimulated: EURA baseline: 5.0 (3.5–7.0), follow up: 5.0 (3.0–8.6); CRA baseline: 5.5 (3.8–8.0), follow up: 5.0 (4.6–8.3)	n/a	n/a
Almasi et al., 2021 * [23]		DMFT: * “dental caries were more prevalent in RA patients, but severe dental caries occur more in control group”	* PI: “dental plaques in RA patients are significantly more than the others”; good oral hygiene: RA: 27.1%, C: 29.7%; moderate oral hygiene: RA: 50%, C: 43.2%; low oral hygiene: RA: 45.8%, C: 27.1%; GI: RA: 55.4% gingivitis, C: 64.4% gingivitis	yes *	2.880 ± 0.99 in RA patients	27.40 ± 15.64	1+: 27.1%, 2+: 45.7%, 3+: 27.1%	n/a	n/a	n/a
Kim et al., 2019 [24]	RA: 22.6 ± 0.6; C: 25.7 ± 0.05	Prevalence: caries in permanent teeth: RA: 0.5%, C: 35.7%	n/a	n/a	n/a	n/a	n/a	n/a	n/a	n/a
Kroese et al., 2022 [25]	Remaining teeth: early rheumatoid arthritis: 27 (24.8–28), risk of RA: 27 (25–28), control: 27.5 (25–28)	DMFT early rheumatoid arthritis: 12.8 (7.0), risk of RA:12.2 (5.9), control: 11.0 (6.5)	n/a	n/a	n/a	n/a	>5.0 kU/L = seropositive	n/a	n/a	n/a
Martinez-Martinez et al., 2019 [10]	Missing teeth: RA: 3.9 ± 3.35 (0–15), Control: 4.0 ± 3.88 (0–16)	DMFT: RA: 13.02 ± 4.99, C: 14.84 ± 5.52, DT: RA: 5.79 ± 3.98, C: 3.88 ± 4.05	n/a	n/a	n/a	n/a	n/a	n/a	n/a	Total of cariogenic bacteria: RA: 3.3 × 10^8^ ± 8.2 × 10^8^ (6483.4 − 4 × 10^9^), control: 4.6 × 10^8^ ± 3.1 × 10^9^ (677.3 − 2 × 10^10^); S. mutans: RA: 5.9 × 10^7^ ± 1.7 × 10^8^ (41.72 − 8.9 × 10^8^), control: 1.5 × 10^5^ ± 4.9 × 10^5^ (66.36 − 2.8 × 10^6^); S. sobrinus: RA: 5.9 × 10^8^ ± 1.8 × 10^9^ (0−7.8 × 10^9^), control: 9.5 × 10^8^ ± 5.6 × 10^8^ (0−3.8 × 10^8^)
Silvestre-Rangil et al., 2016 [26]	n/a	DMFT: RA: 11.84 ± 6.658, C: 10.56 ± 6.621	Plaque index: RA: 1.60 ± 0.579, C: 1.07 ± 0.594	n/a	n/a	n/a	n/a	mL/5 min: RWS (resting whole saliva): control 2.15 ± 1.545, RA 1.45 ± 0.765; SWS (stimulated whole saliva): control 4.564 ± 2.7947, RA 3.811 ± 2.2043; SPS (stimulated parotid saliva flow): control 1.187 ± 1.0838, RA 0.489 ± 0.5298	n/a	n/a
Juan et al., 2022 [27]	n/a	Prevalence: RA: 55.9%, C: 51.5%	n/a	n/a	n/a	n/a	n/a	n/a	n/a	n/a
Mok et al., 2022 [28]	Remaining teeth: (sample size n = 238) 24.8 ± 7.1	(Sample size n = 238) DMFT: 12.8 ± 8.1 (97.9%); DT: 0.8 ± 1.5 (34%)	Plaque index: 0.73 ± 0.19 (sample size n = 183)	n/a	n/a	n/a	74.8% +	n/a	n/a	n/a
Sánchez-Medrano et al., 2021 [29]	Missing teeth: RA: 2 ± 2 (0–9), Control: 2 ± 3 (0–11)	DMFT index: RA 0.51 ± 0.14 (healthy teeth: 13 ± 5 (7–21), D: 9 ± 4 (1–19), M: 2 ± 2 (0–9), F: 2 ± 4 (0–13)), control: 0.44 ± 0.17 (0.14–0.67) (healthy teeth: 15 ± 5 (7–24), D: 5 ± 4 (0–17), M: 2 ± 3 (0–11), F: 5 ± 5 (0–14)); FS-T index: RA: 13 ± 4 (7–21), control: 15 ± 5 (9–24); TNI index: RA: 102 ± 4 (100–111), control: 85 ± 42 (0–114); CI index: RA: 17 ± 30 (0–92), control: 46 ± 40 (0–81)	n/a	n/a	RA: 15% with moderate disease activity (DAS28-ESR: 3.2–≤5.1), 85% with high disease activity (DAS28-ESR: >5.1) [DAS28 = 5.7 ± 0.93 (3.6–7.6)]	yes	n/a	Total salivary flow: RA 5.2 ± 2.0 (1.50–9.0), control 11.6 ± 1.5 (8.5–14); salivary flow: RA 1.0 ± 0.4 (0.30–1.8), control 2.3 ± 0.3 (1.7–2.8)	RA: 7.0 ± 0.4 (6.56–8.1), control: 7.4 ± 0.2 (7.0–7.2)	n/a
de Pablo et al., 2007 [30]	Missing teeth: RA: 20 ±10, Non-RA: 16 ± 11	Decayed surfaces: RA: 2%, non-RA: 4%; decayed or filled surfaces: RA: 21%, non-RA: 24%	n/a	n/a	n/a	n/a	RA: 41% −, 18% +; non-RA: 52% −, 46% +	n/a	n/a	n/a

* No result tables with numerical values available. MT: missing teeth, DT: decayed teeth, FT: filled teeth, DMFT: decayed, missing and filled teeth index, PI: plaque index, GBI: gingiva bleeding index, CPI: community periodontal index, UWS: unstimulated whole saliva, SWS: stimulated whole saliva, n/a: not applicable, SOHI: simplified oral health index.

**Table 3:** Studies which compared caries prevalence between patients with RA and healthy controls.

Author, Year	Number of Participants		Caries Disease Group	Caries Healthy Control Group	Significant Difference between Disease and Control
	RA	Control			
Helenius et al., 2005 [18]	24	24	Prevalence: 75% in patients with missing teeth, 17% in patients with caries at clinical examination	Prevalence: 53% in patients with missing teeth, 22% in patients with caries at clinical examination	no
Gonzales-Chavez et al., 2019 a [21]	30	30	Missing: 6.90 ± 5.77; cavities: 13.46 ± 5.48, slight cavities: 4.30 ± 4.39, moderate cavities: 6.83 ± 5.25, advanced: 2.33 ± 3.45	Missing: 3.03 ± 2; cavities: 4.90 ± 6.55, slight cavities: 2.33 ± 3.91, moderate cavities: 1.96 ± 3.48, advanced cavities: 0.6 ± 1.88	yes
Mehdipour et al., 2022 [14]	45	45	DMFT: 18.87 ± 6.76, DT: 3.84 ± 3.90	DMFT: 11.11 ± 5.90, DT: 1.93 ± 1.86	yes
Äyräväinen et al., 2018 [13]	81 (EURA: n = 53, CRA: n = 28)	43	DMFT: EURA: baseline: 19 (12–23), follow up: 20 (13–24); CRA: baseline: 19 (12–23), follow up: 19 (12–23),	DMFT: 17 (10–21)	yes
Almasi et al., 2021 * [23]	118	118	DMFT: * “dental caries were more prevalent in RA patients, but severe dental caries occur more in control group”	*	yes
Kim et al., 2019 [24]	157	20,140	Prevalence: caries in permanent teeth: 0.5%	Prevalence: caries in permanent teeth: 35.7%	no
Kroese et al., 2022 [25]	150	50	DMFT early rheumatoid arthritis: 12.8 (7.0), risk of RA: 12.2 (5.9)	DMFT: 11.0 (6.5)	no
Martinez-Martinez et al., 2019 [10]	80	80	DMFT: 13.02 ± 4.99, DT: 5.79 ± 3.98	DMFT: 14.84 ± 5.52, DT: 3.88 ± 4.05	DMFT: no, DT: yes
Silvestre-Rangil et al., 2016 [26]	73	73	DMFT: 11.84 ± 6.658	DMFT: 10.56 ± 6.621	no
Juan et al., 2022 [27]	1337	13,370	Prevalence: 55.9%	Prevalence: 51.5%	yes
Sánchez-Medrano et al., 2021 [29]	13	16	DMFT: 0.51 ± 0.14, DT: 9 ± 4	DMFT: 0.44 ± 0.17, decayed: 5 ± 4	DMFT: no, DT: yes
de Pablo et al., 2007 [30]	103	4358	Decayed surfaces: 2%, decayed or filled surfaces: 21%	Decayed surfaces: 4%; decayed or filled surfaces: 24%	Decayed surfaces: yes, decayed or filled surfaces: no

* No result tables with numerical values available. n/a: not applicable.

**Table 4:** Findings of the quality assessment according to [17].

Item	(1) Define the Source of Information (Survey, Record, Review)	(2) List Inclusion and Exclusion Criteria for Exposed and Unexposed Subjects (Cases and Controls) or Refer to Previous Publications	(3) Indicate Time Period Used for Identifying Patients	(4) Indicate Whether or Not Subjects Were Consecutive If Not Population-Based	(5) Indicate If Evaluators of Subjective Components of Study Were Masked to Other Aspects of the Status of the Participants	(6) Describe Any Assessments Undertaken for Quality Assurance Purposes (e.g., Test/Retest of Primary Outcome Measurements)	(7) Explain Any Patient Exclusions from Analysis	(8) Describe How Confounding Variables Were Assessed and/or Controlled.	(9) If Applicable, Explain How Missing Data Were Handled in the Analysis	(10) Summarize Patient Response Rates and Completeness of Data Collection	(11) Clarify What Follow-Up, If Any, Was Expected and the Percentage of Patients for Which Incomplete Data or Follow-Up Were Obtained	Total Score
Helenius et al., 2005 [18]	yes	no	no	yes	no	no	yes	no	n/a	yes	n/a	4
Arneberg et al., 1992 [19]	yes	no	no	yes	no	yes	yes	yes	yes	yes	n/a	7
Descamps et al., 2020 [20]	yes	no	yes	yes	no	no	yes	no	n/a	yes	n/a	5
Gonzales-Chavez et al., 2019 a [21]	yes	yes	yes	yes	no	no	yes	no	n/a	yes	n/a	5
Mehdipour et al., 2022 [14]	yes	yes	no	yes	no	no	yes	no	n/a	yes	n/a	5
Gonzales-Chavez et al., 2019 b [22]	yes	yes	no	yes	no	no	yes	no	n/a	yes	n/a	5
Äyräväinen et al., 2018 [13]	yes	no	yes	yes	yes	no	yes	no	yes	yes	yes	8
Almasi et al., 2021 [23]	yes	yes	no	yes	no	no	yes	no	n/a	yes	n/a	5
Kim et al., 2019 [24]	yes	yes	yes	yes	no	yes	yes	yes	yes	yes	n/a	9
Kroese et al., 2022 [25]	yes	yes	yes	yes	no	no	no	no	yes	yes	n/a	6
Martinez-Martinez et al., 2019 [10]	yes	yes	no	yes	no	no	yes	no	n/a	yes	n/a	5
Silvestre-Rangil et al., 2016 [26]	yes	yes	yes	yes	no	no	yes	no	n/a	yes	n/a	6
Juan et al., 2022 [27]	yes	yes	yes	yes	no	no	yes	no	n/a	yes	n/a	6
Mok et al., 2022 [28]	yes	yes	yes	yes	no	no	yes	no	n/a	yes	n/a	5
Sánchez-Medrano et al., 2021 [29]	yes	yes	yes	yes	no	no	yes	no	n/a	yes	n/a	6
de Pablo et al., 2007 [30]	yes	no	yes	yes	no	no	yes	no	n/a	yes	n/a	4

## Data Availability

All data generated or analyzed during this study are included in this published article.

## Abbreviations

RA	rheumatoid arthritis
EURA	early untreated rheumatoid arthritis
CRA	chronic rheumatoid arthritis
CRP	C-reactive protein
DAS-28	disease activity score
ESR	erythrocyte sedimentation rate
RF	rheumatoid factor
DT	number of decayed teeth
DMFT	decayed, missing and filled teeth index
FT	number of filled teeth
GI	gingival index
MT	number of missing teeth
PI	plaque index
